# Emerging indocyanine green-integrated nanocarriers for multimodal cancer therapy: a review

**DOI:** 10.1039/d1na00059d

**Published:** 2021-04-15

**Authors:** Karunanidhi Gowsalya, Vellingiri Yasothamani, Raju Vivek

**Affiliations:** Bio-Nano Therapeutics Research Laboratory, Cancer Research Program (CRP), School of Life Sciences, Department of Zoology, Bharathiar University Coimbatore-641 046 India vivekr@buc.edu.in

## Abstract

Nanotechnology is a branch of science dealing with the development of new types of nanomaterials by several methods. In the biomedical field, nanotechnology is widely used in the form of nanotherapeutics. Therefore, the current biomedical research pays much attention to nanotechnology for the development of efficient cancer treatment. Indocyanine green (ICG) is a near-infrared tricarbocyanine dye approved by the Food and Drug Administration (FDA) for human clinical use. ICG is a biologically safe photosensitizer and it can kill tumor cells by producing singlet oxygen species and photothermal heat upon NIR irradiation. ICG has some limitations such as easy aggregation, rapid aqueous degradation, and a short half-life. To address these limitations, ICG is further formulated with nanoparticles. Therefore, ICG is integrated with organic nanomaterials (polymers, micelles, liposomes, dendrimers and protein), inorganic nanomaterials (magnetic, gold, mesoporous, calcium, and LDH based), and hybrid nanomaterials. The combination of ICG with nanomaterials provides highly efficient therapeutic effects. Nowadays, ICG is used for various biomedical applications, especially in cancer therapeutics. In this review, we mainly focus on ICG-based combined cancer nanotherapeutics for advanced cancer treatment.

## Abstract 2

Nanotechnology is a branch of science dealing with the development of new types of nanomaterials by several methods.

## Graphical abstract

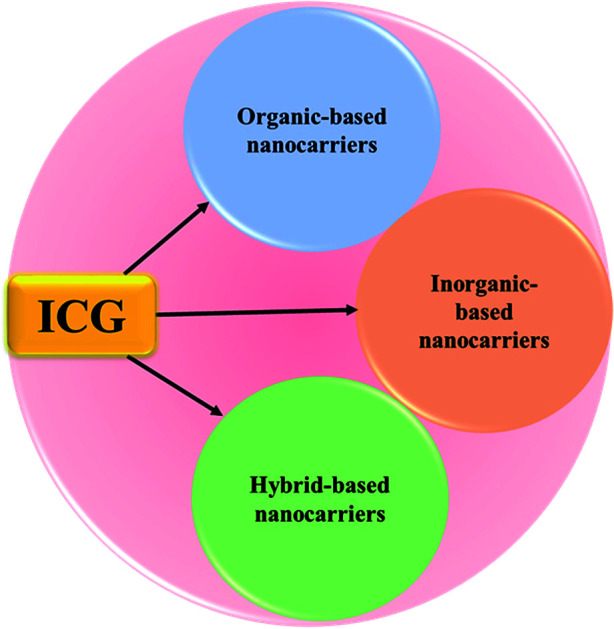

## 1. Introduction

Cancer is one of the deadliest diseases worldwide and causes uncontrolled growth of cells anywhere in our body resulting in tumors, thereby damaging the immune system, and in turn, turning out to be fatal.^1^ These abnormal cells are termed malignant cells, tumor cells, or cancer cells. Cancer cells destroy the immune system of the body hindering its regular function. The abnormal growth of cells causes tumors, while some types of cancers such as leukemia do not form tumors.^2^ Cancer cells may infiltrate into normal body tissues. The abnormal cells comprise the cancer tissue and are additionally identified by the name of the respective tissue from which the abnormal tissue originated, for example, lung cancer, breast cancer, colorectal cancer, and skin cancer. Moreover, cancer cells have some unique features such as abnormal and uncontrollable cell growth and division, absence of proper signals, avoidance of programmed cell death, a limitless number of cell divisions, promotion of blood vessel construction, invasion of tissue, and formation of metastases.^3^ Anything that can cause a normal body cell to develop abnormally causes cancer, *e.g.* some cancer causative agents are chemical compounds, ionizing radiation, and some pathogens. Furthermore, genetic factors also contribute to the development of cancer. The treatment of cancer is limited to chemotherapy, radiation, and surgery.^4^ Cancer symptoms and signs are not unique but different cancers have different types of symptoms such as fatigue, weight loss, pain, skin changes, and changes in lumps. Malignant and benign lesions have some differentiating features, *e.g.* rapid growth, increased cell turn-over, invasive growth, metastases, and vascular, or lymphatic channel invasion are observed in malignant lesions.^5^ Immunotherapy is defined as a type of cancer treatment that prevents the disease with substances that stimulate the immune response and reinitiate the autoimmune response for fighting cancer cells. In clinical cancer management, immunotherapy has an important research value.^6^ Recently, it has been reported that cancer immunotherapy attacks tumors by stimulating or tuning the immune system, and it could inhibit tumor metastasis and recurrence by inducing systemic antitumor immune response.^7,8^ Nanotechnology is the study and application of extremely small structures and is conducted on the nanoscale, which is 10^−9^ meters. Besides, nanotechnology affords the base for designing reasonable drug carriers. Due to their biocompatibility, studies greatly focus on high specific surface area nanocarrier-based delivery systems.^9^ Nanomedicine is defined as medical application of nanotechnology, and it aims to use the properties of nanomaterials for disease diagnosis and to cure diseases at the molecular level. Materials with the particle size ranging from 1 to 100 nm in one or more external dimensions are commonly termed nanomaterials. At first, nanomaterials were used as diagnostic or therapeutic agents through biological barriers. Later, nanomaterial surfaces were coated with polymers or biorecognition molecules for improving nanomaterials’ biocompatibility and used in targeted drug delivery systems to tumor sites.^10^ Nanotherapeutics have the potential to actively target tumors, which increases treatment efficacy while limiting side effects. Chemotherapy is defined as a drug treatment that uses powerful chemicals to kill tumor cells directly. Also, chemotherapy is a major therapeutic approach for cancer treatment which may be used separately or combined with other forms of therapy.^11^ Traditional chemotherapeutics have some unfavorable effects such as poor stability and aqueous solubility. Chemo drugs kill cancer cells, inhibit cancer cell growth, and trigger apoptosis in cancer cells. In many cases, the cancer cells are not eradicated, but they are controlled and managed.^12^ Nanoparticles (NPs) are ultrafine particles that are usually defined as particles of matter. NPs exist in different forms, and based on their properties, shapes, and sizes they are classified into different classes. Also, NPs exist in a variety of shapes including spheres, discs, hemispheres, cylinders, cones, tubes, and wires, and they can also be hollow, porous, or solid. The different groups of NPs include organic NPs, inorganic NPs and hybrid NPs. NPs vary in size from 1 to 100 nanometers (nm) with a very large surface area compared to their volume, and hence they are frequently capable of reacting very quickly. Moreover, NPs possess unique physical and chemical properties. Drug-loaded NPs are widely used in the treatment of various cancer types.^13^ In addition, NPs show many advantages in drug delivery systems but there are still some limitations such as poor oral bioavailability, inadequate tissue distribution, instability in circulation, and toxicity. One of the main advantages of NPs is that their size is tunable. Also, NPs must have the ability to remain in the bloodstream for a considerable amount of time without being eliminated for effective drug delivery into the targeted tumor tissue.^14^ ICG is a water-soluble amphiphilic tricarbocyanine dye that strongly absorbs near-infrared (NIR) light and acts as a photothermal agent. It is negatively charged and belongs to the cyanine family. In 1958, ICG was approved by the United States Food and Drug Administration (US FDA) for medical diagnostic studies.^15^ The short half-life of ICG of 2 to 4 minutes leads to its rapid elimination from the body. Its molecular weight is about 774.96 g mol^−1^ (751.4 Da), and the hydrodynamic diameter of ICG is 1.2 nm.^16^ ICG shows a low incidence of adverse reactions and it exhibits a strong absorption band around 800 nm.^17–19^

**Fig. 1:**
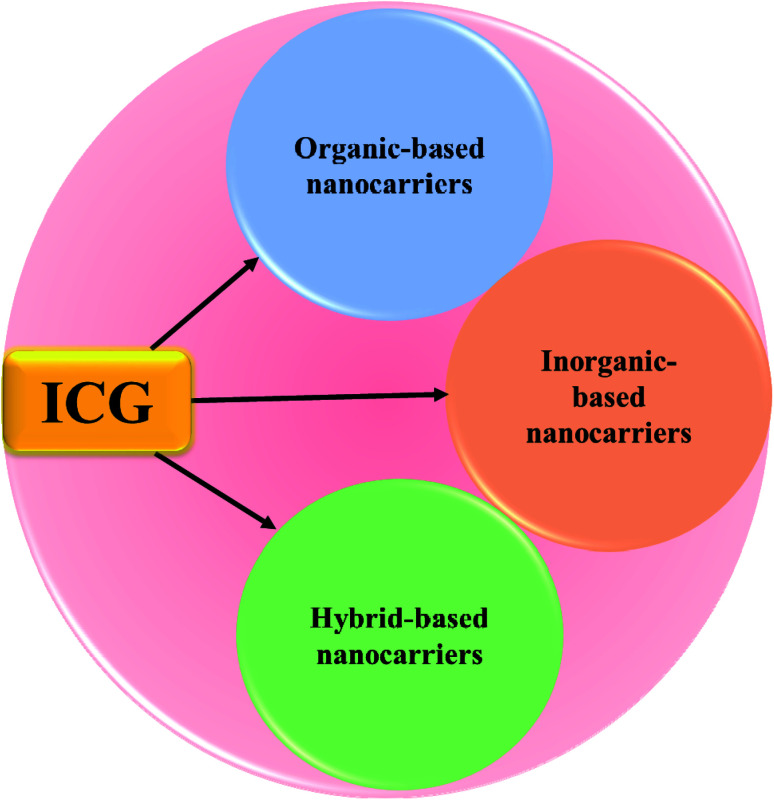
Schematic illustration of ICG-based organic, inorganic, and hybrid nanocarriers.

In this article, we will review the various types of ICG NPs, and discuss their application in cancer theranostics. A huge number of review articles define a clinical diagnostic or therapeutic system of ICG for the treatment of cancer; however, few articles have concentrated on ICG NPs capable of concurrent multimodal imaging and treatment of cancer. Thus, this review is focused on ICG-based formulated nanotherapeutics, such as organic nanomaterials (polymers, micelles, liposomes, dendrimers, protein-based), inorganic nanomaterials (magnetic, gold, mesoporous, calcium, LDH-based), and hybrid nanomaterials (containing both organic and inorganic components), respectively, see Fig. 1.

These ICG-based nanomaterials have highly efficient therapeutic effects. Moreover, the *in vivo* diagnosis of tumors, tumor photothermal therapy, photodynamic therapy of tumors, and theranostics for monitoring biological responses and therapeutic efficacy of the treatment are well discussed. Nowadays, ICG-integrated nanotherapeutics are used for various biomedical purposes, especially in cancer therapeutic applications. In this review, we mainly focus on ICG-based combined nanotherapeutics for advanced cancer treatment. Finally, we address the limitations and future challenges of ICG-based NPs.

## 2. ICG as a versatile agent in cancer therapy

**Fig. 2:**
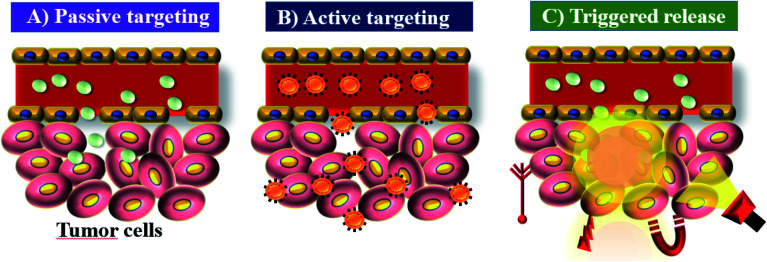
EPR: (A) passive targeting of nanoparticles, (B) active targeting of nanoparticles, and (C) triggered release of nanoparticles under tumor microenvironment conditions.

ICG acts as a fluorescence contrast agent and is used for PTT and photodynamic therapy (PDT). PTT is one of the most efficient therapies that can induce necrosis of malignant tumors with minimal side effects compared to other treatments.^20^ ICG produces singlet oxygen to destroy cancerous cells and tissues through its photosensitizing property. The limited accumulation and photobleaching restrict ICG’s use in PTT. In aqueous solution, ICG is found to form J-aggregates upon heating.^21^ J-Aggregates act as a promising photothermal coupling agent (PCA). PDT is a non-invasive treatment that combines a photosensitizer and an activating light source. Combined treatment with the photosensitizer ICG and NIR light irradiation was initially used for treating skin lesions.^22^ The permissible limit of ICG for clinical use is 0.1–0.5 mg kg^−1^, and it interacts with plasma proteins and acts as an excellent vascular agent for evaluating blood perfusion and lymphatic drainage. Cancer nanomedicine is the application of nanomedicine to the treatment of cancer. This field has seen enormous progress in recent years, but there is still much to achieve. Key concepts in this field are the enhanced permeability and retention (EPR) effect, tumor targeting and accumulation, and the role of nanotechnology in cancer medicine.^13,23^ Nano sized drugs penetrate better into the tumor site through permeable tumor vessels and then they are retained in the tumor bed due to reduced lymphatic drainage. This process is called the EPR effect and it can help to improve the drug uptake into the tumor site, increasing the therapeutic effect,^24^ see Fig. 2.

Nowadays a variety of nano vehicles including protein cages, lipid polymers, and human serum albumin (HSA) have been developed as ICG carriers.^25,26^ ICG is a non-toxic agent but it has some limitations as follows: it degrades in aqueous solution, high temperature accelerates its degradation, it has a short circulation time, it easily binds nonspecifically to HSA which results in its rapid clearance by the liver, and it has low fluorescence quantum yields due to internal conversion and photobleaching. ICG has attracted attention in the field of biomedicine due to its phototherapeutic capability under NIR laser irradiation. ICG is an amphiphilic molecule, consisting of a lipophilic polyaromatic polyene group and a hydrophilic sulfonate group.^27^ ICG can interact with lipophilic and hydrophilic molecules and it can form dimers and oligomers at different concentrations in aqueous solution.^28^ ICG was loaded into NPs as they have enhanced photostability, long circulation time, thermal stability, and tumor targeting ability.^29,30^ ICG is also used as a biologically safe photosensitizer and is used in the destruction of tumors. The optical properties of ICG are very sensitive to some factors such as solvent type, concentration, and temperature. In aqueous solution it undergoes some physicochemical degradation, which reduces its absorption and fluorescence.^31,32^ ICG molecules aggregate and bind to plasma proteins including albumin and globulin in the blood, which enhances the fluorescence intensity.^33^ Numerous NP delivery systems have been developed to load ICG to improve its stability and enhance its pharmacokinetic behaviour, resulting in efficient PTT.^34^

## 3. ICG-based organic nanocarriers

Organic NPs are commonly described as solid particles and composed of organic compounds such as lipids and polymers. Nature provides a wide variety of organic NPs such as protein aggregates, lipid bodies, milk emulsions, and more complex organized structures. One of the most important features of organic NPs is that they provide a route for the encapsulation of materials. The main advantages of organic NPs are extraction from renewable sources, reinforcing capability, low energy consumption, high specific mechanical performance, low density, and biodegradability. Some examples of organic NPs are polymers, liposomes, dendrimers, and polymeric micelles. These organic compounds are used in the field of nanotherapeutics, and ICG can be integrated with many types of organic NPs. These organic compounds act as nanocarriers in the field of nanomedicine. Many nanocarriers such as polymers, micelles (made from amphiphilic block copolymers), liposomes (made from lipid bilayers), dendrimers, *etc.* have been designed to transport drugs to the targeted areas. The main goal of nanomedicine is to develop a safe and effective drug carrier that selectively delivers cytotoxic drugs to tumor cells without harming normal cells.^35^ In ICG-based organic nanotherapeutics, we mainly focus on ICG-based polymers, micelles, liposomes, dendrimers, and protein-based NPs.

### 3.1. Polymeric nanocarriers

**Fig. 3:**
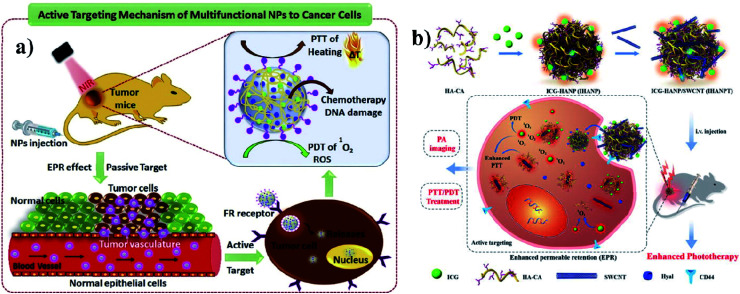
(a) Schematic representation of trimodal therapy, PDT/PTT/chemotherapy, in a human breast cancer model. Reproduced with permission from ref. 37. Copyright 2018, Elsevier. (b) Design and function of the dual-targeted phototherapy agent indocyanine green. Reproduced with permission from ref. 38. Copyright 2016, American Chemical Society.

A polymer is a material made from long and repeating chains of molecules. Polymeric NPs are made from polymers, and ICG can be incorporated and coated with polymers such as PLGA, PLA, and chitosan for nanotherapeutic uses. These polymeric NPs are used as carriers. PLGA possesses a multidrug resistance (MDR) reversal activity independently and it can be used for formulating drugs.^36^ Cancer cells show MDR activity and the cells become resistant to a variety of unrelated drugs in addition to the controlling drug. PLGA is a copolymer of poly(lactic acid) and poly(glycolic acid) and is an FDA-approved biodegradable polymer. It is used to accumulate a drug at the tumor site and the nanocomposite stimulates antitumor activity, with combined properties of PTT and PDT and no chemotherapeutic side effects. After the internalization by the cell, the drug can be released from PLGA dominated by diffusion and it can last for months. PLGA shows good biocompatibility, biodegradability, and controllability in terms of drug release. Vivek and group developed a multifunctional, advanced, targeted nanomedicine with folate-conjugated PLGA (FA–PEG–PLGA–ICG–CBP NPs) for receptor-targeted nano delivery. Folic acid is a targeting ligand for folate-receptor targeted delivery, and the folate receptor is overexpressed in human breast carcinoma cells. Particularly, targeted delivery of dual CBP/ICG loaded NPs provided targeted detection and then CBP interfered with DNA damage and ICG generated singlet oxygen as well as photothermal heat when irradiated with NIR for trimodal PDT/PTT/chemotherapy. In this study, MCF-7 cell lines were used. The antitumor potential was observed at 808 nm, proving its advanced properties for trimodal therapy,^37^ see Fig. 3a. Hyaluronic acid (HA) is a disaccharide polymer that naturally exists in the human body and it has good hydrophilicity. It has been developed as a cancer theranostic agent, because of its tumor-targeting ability and biocompatibility. Moreover, HA can specifically bind to cluster determinant 44 (CD44), a cell surface protein that is highly expressed in cancer stem cells and lymphatic vessels. Endothelial hyaluronic receptor-1 is overexpressed in many types of cancer tissues. The ability of HA NPs to actively and passively target tumors makes them an ideal carrier for the targeted delivery of therapeutic genes, antitumor drugs, and phototherapy agents,^38^ see Fig. 3b.

PLGA is used in drug delivery systems with the simultaneous incorporation of chemotherapeutic agents such as doxorubicin (DOX). DOX is an anthracycline antibiotic drug that can prevent DNA replication by intercalating between base pairs in the DNA helix. DOX is an FDA-approved chemotherapeutic drug for hematological malignancies, many types of carcinomas, and soft tissue sarcomas.^39^ Nanocapsules were made of a PLGA–polymer matrix coated with some ions such as Fe/FeO core–shell nanocrystals (DOX-ICG@Fe/FeO-PPP; PPP is PLGA–PEG–poly(*N*-isopropyl acrylamide) and co-loaded with the chemotherapy drug DOX and the photothermal agent ICG for chemotherapy or PTT. These constructed nanocapsules were used for tumor accumulation and increasing therapeutic ability.^40^ Chen and group formulated the PLGA–ICG–R837 nanocomposite, which is composed of three US FDA-approved agents: PLGA acts as an encapsulating polymer, ICG acts as a NIR agent, enabling photothermal therapy, and finally, imiquimod (R837) is a strong TLR7 agonist in stimulating immune responses. Compared with other therapies, photothermal therapy with immunotherapy shows high efficacy in cancer immunotherapy. Primary tumors were injected with PLGA–ICG–R837 at the tumor site in mice. It could inhibit tumor metastasis and post-spreading of tumors.^41^ ICG-loaded polymers integrated with lipid NPs (INP) were developed for NIR imaging and PTT. Endocytosis and subcellular localization occurred *in vitro* and metabolic distribution and accumulation occurred *in vivo*, and this process was observed through NIR fluorescence.^42^ To prevent the growth of human epidermal growth factor receptor 2 (HER2) expressing breast cancer cells, anti-HER-2-ICG-encapsulated PEG-coated PLGA NPs (HIPP NPs) were prepared for targeted phototherapy. Poly(lactic acid) (PLA) is a biodegradable polymer with good biocompatibility and it is used to encapsulate anticancer drugs. ICG was stabilized by encapsulating into PLA using a coaxial electrospinning method. The main advantages of ICG encapsulation were improvement of its stability and prevention of its rapid degradation in the tumor microenvironment.^43^ Lee and co-workers developed anti-HER2-ICG-DOX-encapsulated PEG–PLGA diblock (PEG-*b*-PLGA) copolymeric NPs (HIDPPNPs) for photochemotherapy and target-specific treatment of HER2-overexpressing breast cancer cells. PEG is an FDA-approved polymer, and is often used to reduce the toxicity and immunogenicity of drug carriers.^44^ In this study, ICG encapsulated and biodegradable PLGA–lipid NPs with folic acid targeting ligands (FA–ICG–PLGA–lipid NPs) were fabricated for *in vivo* photoacoustic molecular imaging. These NPs were evaluated *in vitro* using folate receptor-positive (MCF-7) and negative (A549) cells.^45^ Li and group reported cisplatin (CDDP), which is a platinum anticancer drug used for many solid tumors, and it was approved by the FDA in 1978 for cancer treatment. They developed a CDDP drug delivery system with high encapsulation efficacy. The nanocarrier is made of co-ordination of tellurium-block polymers (PEG–PUTe–PEG) and was loaded with CDDP and ICG concurrently. The nanocarriers selectively released the CDDP at the tumor site, and achieved high antitumor efficacy and reduced side effects.^46^

### 3.2. Micellar nanocarriers

**Fig. 4:**
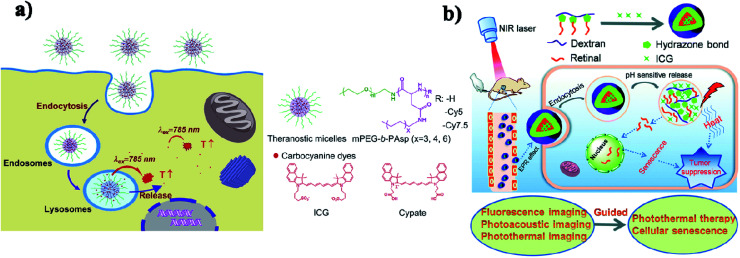
(a) Schematic illustration of theranostic micelles for prolonged cancer imaging and photothermal therapy with carbocyanine dyes. Reproduced with permission from ref. 57. Copyright 2013, Elsevier. (b) Schematic illustration of pH-sensitive loaded retinal/ICG micelles as an all-in-one theranostic agent for multimodal imaging. Reproduced with permission from ref. 54. Copyright 2019, Royal Society of Chemistry.

Micelles are spherical and colloidal NPs that are self-assembled by amphiphilic polymeric molecules and can be used to encapsulate hydrophobic drugs for tumor drug delivery application. Micelles have a particle size between 5 and 100 nm and act as carriers. Phospholipid micelles are used as novel lipid-based carriers for water-insoluble drugs.^47^ Due to their nanoscale size, polymeric micelles can penetrate easily into the tumor vasculature and accumulate passively at tumor sites through the EPR effect.^48^ Based on the noncovalent self-assembly method, ICG was encapsulated into the core of a polymeric micelle [PEG–polypeptide hybrid triblock copolymers of PEG-*b*-poly (l-lysine)-*b*-poly (l-leucine)]. ICG was associated with the hydrophobic core through hydrophobic interaction and also the hydrophilic heads through electrostatic attractive interaction. PEG–PLL–PLLeu–ICG micelles significantly improved the quantum yield and fluorescence stability compared to free ICG. PEG–PLL–PLLeu–ICG has the potential for use in PTT and imaging. In this study, human lung carcinoma cells (H460) were used. Female BALB/c nude mice were used for *in vivo* studies.^49^ Encapsulation of ICG assesses the stability of the micellar formulations and it is used as an indicator of both *in vitro* stability and *in vivo* tumor accumulation. In the solvent evaporation method, ICG was loaded into micelles. The ICG-loaded micelles were evaluated *in vivo* for tumor accumulation by intravenous delivery to a murine xenograft tumor model. The MDA-MB-435 human melanoma cell line was used.^50^ In a simple aqueous-based preparation method, ICG can be incorporated into micelles and delivered into tumor sites, which enhanced early cancer detection. Pluronic F-127 polymeric micelles were constructed for ICG delivery *in vivo*. ICG can be loaded into pluronic micelles at various concentrations. Pluronic micelles and ICG are characterized by measuring micelle size distribution, dye loading efficiency, and both aqueous and thermal stability, to estimate the circulation kinetics and biodistribution of ICG loaded into micelles *in vivo*.^51^ Polystyrene-*alt*-maleic anhydride-*block*-polystyrene-PSMA-*b*-PSTY diblock copolymers make ICG polymeric micelles suitable for application in NIR diagnostic imaging. This system has the potential to greatly improve NIR diagnostic imaging in breast cancer detection by increasing the stability of ICG for formulation/administration and by providing a means to deliver ICG into the target tumor tissue. Polymeric micelles prevent ICG’s degradation and the micelles' size and stability are suited for passive targeting of ICG-encapsulated micelles to the tumor site.^52^ ICG can be encapsulated in the micelles with folic acid for photodynamic theranostics and they were prepared by a thin-film hydration method. The FA-ICG-micelles are initially in the “OFF” state with no fluorescence signal or phototoxicity, but in cellular degradative environments, they become highly fluorescent and phototoxic, and accumulate at the tumor site.^53^ Zhu and group reported pH-sensitive loaded retinal/ICG micelles as an all-in-one agent for cellular senescence–photothermal synergistic therapy to treat cancer. Amphiphilic dextran retinal (DR) conjugates were synthesized using hydrazine bonds. ICG was loaded into the hydrophobic core of DR conjugates by a dialysis method to form the DR–ICG micelles. DRI micelles significantly suppress tumor growth under laser irradiation,^54^ see Fig. 4b.

In another case, ICG was loaded into active targeting micelles for simultaneous tumor NIR imaging and PTT. These micelles displayed excellent biocompatibility and stable NIR optical properties. After intravenous injection, the micelles effectively targeted the keratin forming tumor cell line HeLa (KB) and realized long-term tumor imaging with high contrast resolution. The micelles exhibited excellent photothermal properties and achieved successful PTT in KB tumor-bearing mice.^55^ Hydrophobic ICG was encapsulated in micelles with hyaluronic acid (HA–ICG) for CD44 receptors and used in NIR and photoacoustic imaging (PAI) of tumors. They can accumulate into the tumor site. Squamous cell carcinoma (SCC-7) and mouse embryonic fibroblast (NIH-3T3) cell lines were used for this study. BALB/c nude mice were used for *in vivo* studies.^56^ Micelles assembled with carbocyanine dyes were used for improved NIRF (near-infrared fluorescence) cancer imaging. A polyaspartamide based micellar system has multiple advantages including small size, high loading capacity, good stability, sustained release, and enhanced cellular uptake. Concurrently, the micelles further generate a superior photothermal effect on cancer cells and achieve efficient photothermal efficacy upon photoirradiation,^57^ see Fig. 4a.

### 3.3. Liposome nanocarriers

**Fig. 5:**
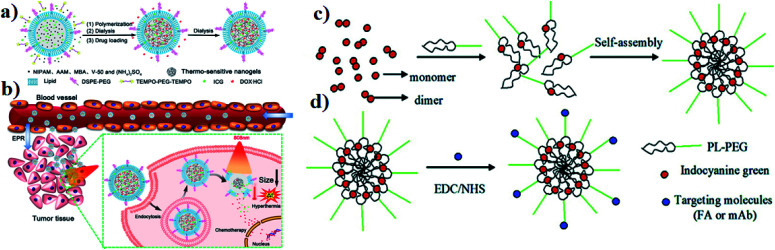
(a) Schematic illustration of DI-NGs@lipo prepared by *in situ* polymerization within a liposome template. (b) NIR-induced hyperthermia triggered intracellular DOX release for synergistic chemo-photothermal therapy. Reproduced with permission from ref. 59. Copyright 2018, American Chemical Society. (c) Self-assembly process of the ICG–PL–PEG probe. (d) Targeted modification of the ICG–PL–PEG probe. Reproduced with permission from ref. 60. Copyright 2011, American Chemical Society.

Liposomes act as nanocarriers, and they are biocompatible and produced using extremely cheap raw materials such as soybean oil, lecithin, *etc.* Doxil (DOX-containing PEGylated liposomes) was the first approved therapeutic liposomal delivery vehicle, and liposomes have been proposed as drug carriers in cancer therapy.^58^ Liposomes can be incorporated with ICG for an effective drug delivery system. In an *in situ* polymerization within a liposome, a template was designed to prepare liposome-coated poly(*N*-isopropylacrylamide-*co*-acrylamide) (P(NIPAM-*co*-AAM)) nanogels, capable of encapsulating the NIR dye ICG and DOX, and are denoted as DI-NGs@lipo. ICG-loaded biodegradable folate lipid PLGA NPs are used in both *in vitro* and *in vivo* treatment. The DI-NGs@lipo composite was taken up by 4T1 murine breast cancer cells through endocytosis, and the cytotoxicity against 4T1 cells was enhanced under NIR irradiation,^59^ see Fig. 5a and b. Further, ICG can be incorporated into phospholipids and PEG (PL–PEG). The interaction between amphiphilic ICG and PL PEG is enhanced as observed by fluorescence spectroscopy. ICG–PL–PEG was used for cell imaging and selective photothermal cell damage. The properties of the ICG–PL–PEG nanoprobe, such as absorption and fluorescence spectra, stability, morphology, and size distribution, were also investigated. Targeting molecules FA and integrin Rvβ3 monoclonal antibodies (mAb) were conjugated on the surface of the ICG–PL–PEG. The novel ICG–PL–PEG probe has several unique features such as a highly stable structure, nontoxicity, great potential for optical imaging, and selective phototherapy,^60^ see Fig. 5c and d.

Anti-tumor liposomal NPs Doxil and poly(d, l-lactide) NPs have been approved by the FDA for clinical and ethical use. ICG and DOX serve as dual-functional agents, show optical imaging capabilities, and are also integrated with PTT.^61^ ICG can physically interact with phospholipids in the liposome membrane, which modifies the stability and quantum yield of ICG as well as the structure and stability of the lipid membrane. ICG can be encapsulated in the aqueous compartment of liposomes. Finally, liposomal ICG acts as an *in vivo* imaging agent.^33^ In another study, ICG is incorporated with liposomes to maximize the photothermal effect and it has great potential for clinical translation in oncology because it is prepared by self-assembly of FDA-approved components such as phospholipids through hydrophobic interactions. Most importantly, ICG-liposomes improved the PTT effect on cancer cells.^27^ Liposomal formulation of ICG shows ideal attributes for lymphatic function imaging. Liposomal ICG showed improved optical properties and prolonged fluorescence stability in solution.^62^ Liposomes are used to formulate ICG and other cyanine dyes for optical imaging and phototherapy, and sometimes ICG liposomes are used to monitor drug delivery into tumor sites. Liposomes are also labeled with ICG (ex: PEGylated liposomes). Liposomal ICG is a highly potent clinical optoacoustic contrast agent and shows good biocompatibility and also biodegradability.^63^ Liposomal ICG can be utilized for rectal tumor imaging and PEGylation of the carriers improved the imaging.^64^ Bhavane and group reported that liposomal ICG elevated the NIR-II fluorescence properties which improved its visualization *in vivo*; in this study, nude mice were used. However, this study demonstrated that comparatively the NIR-II window shows better tissue transparency features than the NIR-I window.^65^ The stability of ICG can be increased when it is encapsulated into liposomes. ICG liposomes have some important advantages such as safety, imaging possibilities, and manufacturing of smaller sizes controlled by liposomes. ICG can be embedded into a bilayer by dissolving with phospholipids in organic solvents.^66^ A liposomal formulation of ICG was developed and evaluated for the treatment of triple-negative breast cancer (TNBC). Liposomal ICG improved the destruction of the cancer tissue. In the TNBC cell line, MDA-MB-468 cells were selected for this study. A xenograft model of nude mice was selected for *in vivo* studies and it enhanced the localized PDT of tumors.^67^ The anti-metastasis drug silibinin and the PDT agent ICG have been shown to simultaneously inhibit tumor cell growth and metastasis *in vitro*. Silibinin and ICG self-assembled into poly(caprolactone) (PCL) lipid NPs (PNs) (ICG–PCL–silibinin) and pluronic copolymer F68 (SIPNs). The 4T1 breast cancer cell line was used for this study and it has a high metastatic capability. The drug release was evaluated *in vitro*^68^. High-density lipoproteins (HDLs) show deep tumor penetrating ability and ICG was used for synergistic phototherapy. Specifically, the HDL protein was conjugated with the tumor-homing iRGD peptide. HDLs and ICG (HDLs–ICG–iRGD) can produce heat for PTT and sufficient reactive oxygen species (ROS) for PDT.^69^ Nanostructured lipid carriers (NLCs) were fabricated with PEG and FA, which act as shielding and tumor-homing segments. Paclitaxel (PTX, an anticancer drug used in many clinical trials) and ICG (NLC–PEG–FA–PTX) were incorporated into the hydrophobic lipid core of NLCs to make a co-delivery system. ICG acts not only as a therapeutic drug but also as a laser responsive agent. NLCs have high biocompatibility and tumor specificity. Squamous cell carcinoma (SCC-7) and mouse embryonic fibroblast (NIH-3T3) cell lines were used for *in vitro* studies. Male BALB/c nude mice and New Zealand rabbits were used for *in vivo* studies.^70^ Sorafenib (SF) is a U.S. FDA-approved first-line drug for the systemic treatment of hepatocellular carcinoma (HCC) patients. The use of ICG with SF could improve the anti-cancer efficacy. SF–ICG–liposomes (SILs) used for *in vitro* and *in vivo* studies enhanced PTT and PDT.^71^ Perfluorooctyl bromide (PFOB) is a biocompatible chemical and is integrated with ICG in a nanoliposome structure through a two-step emulsion method. LIP–PFOB–ICG inhibited MDA-MB-231 tumor growth completely *via* intravenous injection through enhanced PDT and PTT synergistic therapy. PFOB has an excellent oxygen-carrying ability, which effectively attenuated tumor hypoxia.^72^ To overcome ICG limitations, ICG was encapsulated in the core of a polymeric micelle, which self-assembled from amphiphilic PEG–polypeptide hybrid triblock copolymers of PEG poly L lysine (PLL) and poly L leucine (PLLeu). PLLeu is the hydrophobic core and PEG is the hydrophilic shell. The ICG is associated with the hydrophobic core through hydrophobic interaction and also with the hydrophilic heads through electrostatic attractive interaction. Compared with free ICG, PEG–PLL–PLLeu–ICG micelles improved the quantum yield and fluorescence stability.^49^ An ICG-containing nanostructure, ICG–PL–PEG, is developed for PTT, which is self-assembled from ICG and phospholipid PEG (PL–PEG).^22^ Wu and his co-workers have synthesized iRGD peptide-functionalized echogenic liposomes (iELPs), which are used to encapsulate methotrexate (MTX) and ICG for improving the therapeutic efficacy.^73^

### 3.4. Dendrimer nanocarriers

**Fig. 6:**
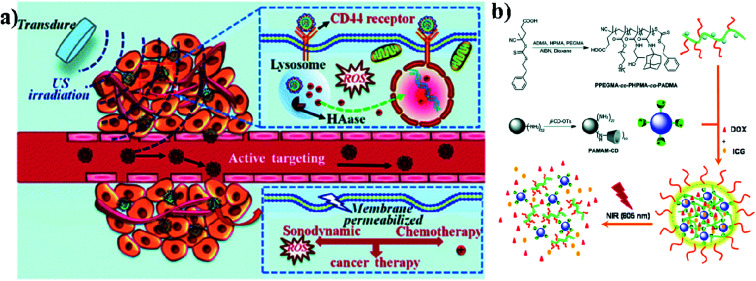
(a) Schematic illustration of sonochemotherapy using HA-modified nanocomposites. Reproduced with permission from ref. 77. Copyright 2019, Royal Society of Chemistry. (b) Schematic illustration of nanogel fabrication from PPEGMA-*co*-PHPMA-*co*-PADMA copolymers and PAMAM–CD dendrimers. Reproduced with permission from ref. 78. Copyright 2015, Royal Society of Chemistry.

Biodegradable dendrimers are non-toxic, non-immunogenic, and preferably biocompatible. These unique features make dendrimers promising alternative agents to traditional linear polymers in the design of drug delivery systems and they may generate an EPR effect during cancer therapy. Acetylated polyamidoamine (PAMAM) dendrimers can successfully prevent the formation of cross-linking between PAMAM and ICG molecules. Acetylated dendrimers also improve the photostability of ICG molecules in aqueous solutions and efficiently decrease the formation of ICG J-aggregates.^74^ Dendrimers are also used in imaging applications, PDT, and drug delivery systems. Conjugation of dendrimer branches with PEG or polyethylene oxide (PEO) can be used for prolonging blood circulation. And dendrimers are also used as therapeutic agents instead of carriers.^75^ PAMAM has high buffer capacities for endo-lysosomal escape. These positive nanocarriers can deliver anticancer drugs into tumor cells through endocytosis. The *in vivo* distribution of free ICG and ICG/PAMAM-n2 PF68 was studied, and at 2 h, free ICG quickly accumulated in the liver. As for the ICG-loaded conjugates, the tumor showed the strongest fluorescence. The distribution of ICG changed after encapsulation by PAMAM-n2 PF68.^76^ Wu and group reported that sonochemotherapy is a strategy for inhibiting tumors. In this study, HPCID was developed, which was composed of hyaluronic acid (HA), carboxyl-terminated PAMAM dendrimer, fluorochrome ICG, and DOX hydrochloride. At first, the amino-terminal group of PAMAM was converted into the carboxyl group with the help of succinic anhydride (SA), to decrease the strong positive charge on PAMAM and make the carboxylate terminated PAMAM dendrimer (PCH). After that, fluorochrome ICG was encapsulated into the PCH and loaded with DOX hydrochloride by electrostatic adsorption to formulate ICG@PCH@DOX (PCID). To improve the cellular internalization by tumor cells, HA was modified on the surface of the PCID NPs. Ultrasonic stimulation improved intratumoral drug diffusion and produced ROS from ICG, to achieve highly effective sonochemotherapy in a breast tumor xenograft mouse model,^77^ see Fig. 6a. Nanogels (NGs) were prepared by supramolecular self-assembly from the adamantine (AD)-conjugated copolymer poly[poly(ethylene glycol)monomethyl ether methacrylate]-*co*-poly(2-hydroxypropyl)-methacrylamide-*co*-poly(*N*-adamantan-1-yl-2methacrylamide) (PPEGMA-*co*-PHPMA-*co*-PADMA) and a β-cyclodextrin (β-CD)-functionalized poly(amidoamine) (PAMAM) dendrimer based on interaction of the AD and β-CD moieties. Finally, ICG and DOX can be encapsulated into NGs (DINGs–DOX–ICG nanogels) with high encapsulation efficiencies (EEs) due to the electrostatic interactions between PAMAM, DOX, and ICG. The encapsulated DOX can be released conveniently. The HepG2 cell line was used for *in vitro* cytotoxic studies and CD-1 (ICR) mice were used for *in vivo* studies. DINGs showed high accumulation under *in vivo* conditions in tumor tissues and significant tumor growth suppression under 805 nm NIR irradiation,^78^ see Fig. 6b.

Tsuchimoci and co-workers developed PAMAM coated silica NPs loaded with ^99m^Tc and ICG (PAMAM–^99m^Tc–ICG) and conducted animal studies to test the probability and utility of the dual-modality imaging system. For *in vivo* studies male Wistar rats were used.^79^ The small organic fluorescent dye ICG can be loaded into a non-cytotoxic polyacylthiourea dendrimer (PD) for optical imaging. PD also acts as a chelator-free precursor for radiolabeling with ^64^Cu for positron emission tomography (PET). This integrated nanomedicine gives a highly versatile platform to develop multimodal theranostic agents.^42^ For achieving dual-modality imaging of HER2-overexpressing cancer cells, Yamaguchi and co-workers developed PAMAM-based functionalized silica NPs and used them for multimodal imaging. The developed functionalized silica NPs were loaded with technetium-99m (^99m^Tc) and ICG for targeting and imaging HER2-expressing cells. These dual-imaging probes were tested on HER2-overexpressing breast carcinoma cells. *In vivo* imaging was examined in breast tumor xenograft mouse models. Comparatively, SK-BR3 (HER2 positive) cells show stronger NIR fluorescence signals than MDA-MB231 (HER2 negative) cells.^80^

### 3.5. Protein nanocarriers

**Fig. 7:**
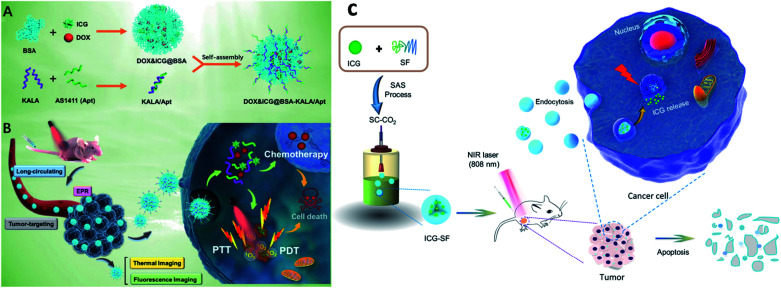
Schematic illustration of (A) preparation of DOX&ICG@BSA-KALA/Apt nanoparticles and (B) the theranostic process based on DOX&ICG@BSA-KALA/Apt nanoparticles. Reproduced with permission from ref. 83. Copyright 2019, American Chemical Society. (C) Schematic illustration showing the outline of preparation of ICG-SF nanoparticles by the SAS process and dual-triggered cancer therapeutics. Reproduced with permission from ref. 84. Copyright 2018, American Chemical Society.

Proteins come under the class of natural biomolecules and have the unique ability to deliver peptides, RNA, and chemotherapeutic drugs such as hydrophilic and hydrophobic drugs. Due to their ability to deliver various therapeutic drugs, proteins are used as delivery carriers. Proteins such as BSA, HSA, and SF are used to deliver drugs into the targeted regions for cancer treatment. In this work, BSA NPs were loaded with artemisinin (ART), which is a chemotherapeutic agent, and it was covalently conjugated with ICG and the tumor-specific arginine–glycine–aspartic acid (RGD) peptide, for imaging-guided and chemo phototherapy. The prepared RGD–ICG–BSA–ART (IBA) NPs have great biocompatibility, water stability, photostability, and photothermal effect. RGD–IBA NPs can concurrently produce hyperthermia and reactive oxygen species (ROS) to destroy tumor cells. The human oral epidermal carcinoma KB cell line and female BALB/c mice were used in this study.^81^ A composite of Pd corolla NPs, HSA, and ICG (PdCs–HSA–ICG) was developed for cancer PTT, PDT, and combination therapy. Pd corollas have NIR photothermal conversion efficiency. They were first prepared and modified with HSA and ICG to obtain the PdCs–HSA–ICG nanocomposite, which has great potential and acts as a photosensitive agent for cancer phototherapy. Female ICR mice were used and human HeLa cell lines were cultured and treated under 808 nm NIR irradiation.^82^ Xu and group developed a DOX–ICG–BSA–KALA–Apt NP nanocomposite. DOX and ICG were self-assembled with BSA molecules to form nanosized DOX–ICG–BSA particles. A tumor-targeting aptamer (AS1411) and a cell-penetrating peptide (KALA) were introduced on DOX–ICG–BSA NPs through electrostatic interaction. ICG is used as a NIR contrast agent, and it also exhibits phototherapeutic effects. The MCF-7 cell line was used and cells were treated under 808 nm NIR irradiation. This study provides a promising strategy to develop a protein-based nano theranostic system for tumor targeting multimodal diagnosis and therapy,^83^ see Fig. 7A and B. Chen and co-workers developed ICG encapsulated silk fibroin (ICG-SF) NPs by supercritical fluid technology (SCF). These NPs have good photothermal stability, show pH-responsive release of ICG from SF especially in the tumor environment, and NIR light at 808 nm enhances their PTT effect. ICG-SF NPs have the capability of disturbing tumor cells under light-induced hyperthermia,^84^ see Fig. 7C.

In a recent study by Pham and co-workers, the authors prepared albumin NPs with antitumor efficiencies through dual advantages of hyperthermia and a chemotherapeutic agent. Therefore, the authors prepared albumin NPs containing ICG and Cur, termed ICG–BSA–Cur NPs for ablation of tumors based on hyperthermia. Curcumin is a natural compound, derived from turmeric, and a potent chemopreventive and chemotherapeutic agent. Finally, these NPs were analyzed with neuroblastoma N2a cells for *in vitro* studies and xenograft nude mice were used for *in vivo* studies.^85^ Sheng and group developed HSA and ICG NPs (HSA–ICG NPs) by intermolecular disulfide conjugations. Further, these NPs effectively induced ROS and hyperthermia for PDT/PDT treatments under NIR laser irradiation. The tumor was completely suppressed and no recurrence was observed after intravenous injection of NPs.^86^

## 4. ICG-based inorganic nanocarriers

Inorganic NPs are made from inorganic elements such as metals, gold, iron oxide, and calcium phosphate. Mainly, inorganic NPs consist of two parts, namely a core and a shell (organic polymers or metals), which protects the core from chemical interactions or serves as a substrate for conjugation with biomolecules. These inorganic NPs are more stable and have higher biocompatibility than organic NPs. Moreover, inorganic NPs are some of the most widely studied materials due to their unique physical and chemical properties that originate from their nanoscale dimensions. Various NP probes for bioimaging were developed using their magnetic, X-ray attenuation, and optical properties, for example, magnetic NPs (*e.g.*, superparamagnetic iron oxide NPs). Further, inorganic NPs still have some drawbacks, and as a result, only very few NP probes are approved for clinical use.^87^ Based on their optical signals many nanomaterials have been constructed as biosensors such as calcium phosphate, dye-doped silica NPs, and gold NPs. ICG can be incorporated into some inorganic materials such as magnate, gold, mesoporous silica, calcium phosphate, and LDH based nanocarriers. These inorganic NPs are used to encapsulate drugs and also act as carriers in targeted drug delivery systems. Nonporous silica NPs, mesoporous silica NPs, quantum dots, gold NPs, and magnetic NPs are used as therapeutic agents in sensing and drug delivery.^88,89^

### 4.1. Magnetic nanocarriers

**Fig. 8:**
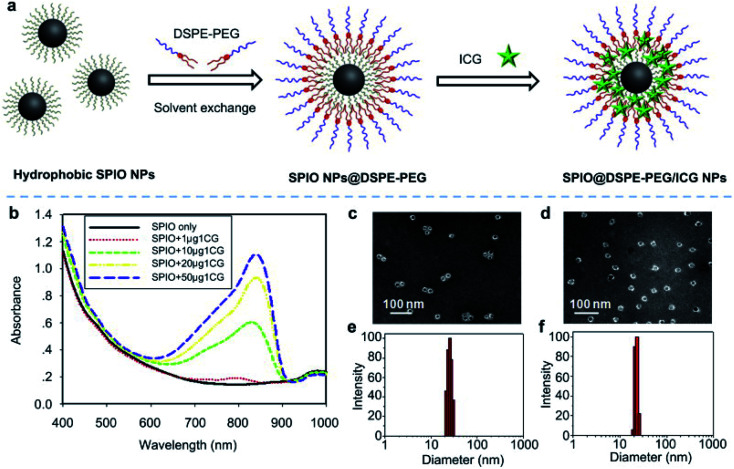
(a) A schematic illustration of SPIO@DSPE-PEG/ICG NP preparation. (b) UV absorbance after treatment with different concentrations of SPIO@DSPE-PEG/ICG NPs with or without NIR laser irradiation. (c and d) TEM images of NPs. (e and f) Size distribution pattern of NPs. Reproduced with permission from ref. 91. Copyright 2013, Elsevier.

In ferric materials, magnetic NPs are an important class, and their magnetization can be controlled by an applied magnetic field. Based on actuation, magnetic NPs are attractive in a wide range of applications. Magnetic NPs include Fe_3_O_4_, Fe^2+^, and superparamagnetic NPs.^90^ In this work, superparamagnetic iron oxide NPs (SPIO NPs) act as a T2 contrast agent and are the first nanoparticulate MRI contrast agent to be used clinically. SPIO NPs can be controlled by an external magnetic field. They are also sophisticatedly employed in a magnetically targeted drug delivery system to regulate drug release, minimizing side effects and improving therapeutic efficacy. ICG molecules are loaded into the lipid layer on the surface of SPIO NPs (ICG–SPIO NPs) for photothermal tumor ablation. SPIO NPs loaded with DSPE–PEG 5000 and ICG enhance MR imaging. The HeLa cell line was used with laser irradiation at 808 nm. ICG serves as a NIR fluorescence probe and a strong NIR light-absorbing agent to efficiently convert absorbed light into heat,^91^ see Fig. 8.

Magnetic Prussian blue (PB) NPs have been synthesized using superparamagnetic Fe_3_O_4_ nanocores for magnetically targeted PTT. PB and ICG were integrated for PDT and PTT and activated by NIR irradiation. In aqueous solutions, PB served as a carrier of ICG to prevent its degradation and enhance its stability. ICG with PB NPs (ICG–PB NPs) were used for the magnetically targeted combination of PDT and PTT. Negatively charged ICG was adsorbed onto the positively charged FPP NPs by electrostatic interaction to obtain the final Fe_3_O_4_@PB/PEI/ICG (FPPI) NPs with high drug loading efficiency. This composite was intravenously injected into BALB/c nude mice for *in vitro* studies. The stability of ICG improved after being conjugated onto the Fe_3_O_4_@PB/PEI nanocarrier, which allowed its intra-tumor accumulation. The HeLa cell line was used for *in vivo* studies and 808 nm laser irradiation was used. The PB nanoshell and loaded ICG worked as photo absorbers for PTT, and also served as photosensitizers for PDT.^92^ Combination of Gd and fluorescent ICG encapsulated, comparably low concentrations in SiO_2_ for core–shell structures. GdO- and ICG-loaded IOH-NPs act as a new multimodal contrast agent for use in OI, PAI, and MRI and are distinguished by a non-complex composition and structure, a water-based synthesis, an exceptional load of ICG and GdO, the absence of inert matrices, and improved performance in comparison to conventional ICG and Gd-DTPA in solution. Mouse alveolar macrophage cell line MH-S (CRL-2019, ATCC) was used for *in vivo* studies. C57BL/6 albino mice bred in-house were used for all animal experiments.^93^ Magnetic iron oxide (Fe_3_O_4_) NPs have low toxicity, strong effects on T2 relaxation for magnetic resonance imaging (MRI), and excellent photo absorbing ability. Perfluoropentane (PFP) based PLGA NPs are loaded with Fe_3_O_4_ NPs and ICG (Fe_3_O_4_/ICG@PLGA/PFP NPs) for effective photothermal tumor ablation. They both (Fe_3_O_4_ NPs and ICG) can efficiently convert light into heat through NIR light absorption. Human breast cancer MCF-7 cell line and female BALB/c mice were used for *in vitro* and *in vivo* studies. They enhance the thermal ablation effect in the local tumor area under 808 nm laser irradiation.^94^ SPIO incorporated with the chemotherapeutic drug DOX and ICG produced SPIO–DOX–ICG NPs in the cancer cell membrane (CCM). 808 nm NIR laser irradiation produced a high level of hyperthermia, which effectively antagonized tumor hypoxia in the tumor region by promoting polarization.^95^ Polydopamine (PDA) is a melanin-like biopolymer and PDA NPs have strong NIR absorption for photoacoustic (PA) and MRI dual-modal imaging-guided cancer PTT. Iron ions (Fe^3+^) as an MRI contrast agent were chelated to PDA NPs. ICG coordinated with the surface of PDA NPs to form coordinating NPs of ICG-loaded PDA–iron ions (PDA–Fe^3+^ + ICG NPs) with high optical absorption in the NIR field. They show good biocompatibility, dual-modal imaging, and efficient PTT. BALB/c mice with the subcutaneous 4T1 breast cancer cell line were used as an animal model. The enhanced NIR absorption of PDA–Fe_3_–ICG NPs efficiently kills cancer cells with low laser power density.^32^ The magnetic graphene oxide (mGO) developed by Ocsoy and co-authors and ICG have been separately considered as photothermal agents. An aptamer (APT), ICG, and mGO were combined to form APT–ICG–mGO. With NIR light irradiation, the aptamer (Apt), ICG and mGO exhibit a cooperative effect by generating high local heat reaching 43 °C in 20 seconds. ICG acts as a PDT agent and also produces singlet oxygen under NIR laser irradiation. The combination of PTT and PDT enhances target cancer cell killing efficiency.^96^

### 4.2. Gold nanocarriers

**Fig. 9:**
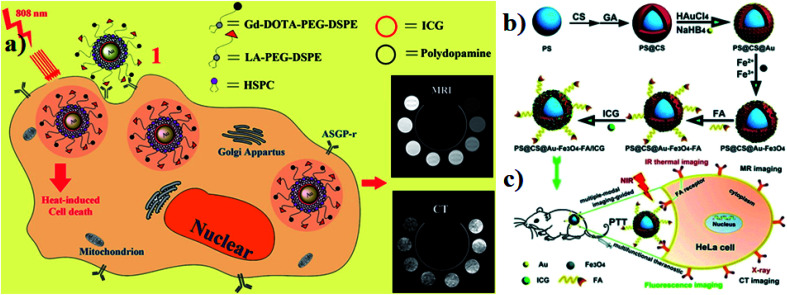
(a) Schematic illustration of synthesis and function of gold nanoparticles with encapsulated ICG. Reproduced with permission from ref. 102. Copyright 2014, American Chemical Society. (b and c) Schematic of the synthesis and application of PS@CS@Au–Fe_3_O_4_–FA/ICG. Reproduced with permission from ref. 103. Copyright 2017, Royal Society of Chemistry.

Gold-based nanomaterials are used as nanocarriers for drug delivery and they have different structures such as nanorods (NRs), nanoshells, nanospheres, and nanocages. AuNPs have unique properties and potential for use in anticancer drug delivery systems that have photothermal cancer treatment agents. Moreover, AuNPs are very small in size and range between 10 and 200 nm. AuNPs are also encapsulated into ICG for an effective nanotherapeutic effect.^97,98^ Guerrini and colleagues developed a gold nanorod (GNR) that adsorbs ICG and citrate reduced gold nanospheres (citrate/GNPs) were used as a model substrate and ICG was selected as a probe. The optical properties of ICG in the bulk solution are very sensitive to dye concentration, ionic strength, pH, and the presence of other solutes. These parameters affect the self-association of ICG monomers into dimers and larger aggregates, which in turn leads to changes in the optical activities.^99^ Hybrid nanocapsules loaded with bovine serum albumin (BSA) capped gold nanoclusters (AuNCs) and ICG were used for dual-modal imaging and effective PTT. RGD peptides are also conjugated on the surface of the hybrid nanocapsules for overexpressing integrin ανβ_3_. U87-MG human glioblastoma cells and MCF-7 breast cancer cells were used for comparative *in vitro* studies. For *in vivo* studies, BALB/c mice were used. The photothermal performance was investigated under 808 nm laser irradiation. The nanocapsules are mainly composed of biodegradable poly mermethoxy PEG–PLGA (mPEG–PLGA) biocompatible for both *in vivo* and *in vitro* drug delivery.^100^ Endoplasmic reticulum (ER) targeting pardaxin peptide-modified, ICG-conjugated hollow gold nanospheres (FAL–ICG–HAuNS) together with oxygen delivering hemoglobin (Hb) liposomes (FAL–Hb-lipo) were designed for reversing hypoxia. Immunogenic cell death (ICD) associated immunogenicity can be evoked by reactive oxygen species (ROS) produced through ER stress. CT-26 mouse colon carcinoma and B16 mouse melanoma cells were used for *in vitro* studies. A double ER targeting strategy realizes both PDT and PTT.^101^ For hepatocellular carcinoma, the theranostic agent lactobionic acid was prepared and used for targeted magnetic resonance imaging/computed X-ray tomography (MRI/CT), dual modal imaging, and PTT. The theranostic agent contains a core–shell structure with AuNPs and PDA as the inner core, ICG as the photothermal therapeutic agent, which is electrostatically adsorbed onto the surface of PDA (AuNPs–PDA–ICG), and lipids modified with gadolinium tetra acetic acid and LA, which are self-assembled on the outer surface of the shell. HepG2 liver cancer cells and HL-7702 normal liver cells were used for comparative *in vitro* studies. Photothermal degradation also occurred under 808 nm laser irradiation,^102^ see Fig. 9a. Wang and group reported that polystyrene–chitosan–gold NPs–Fe_3_O_4_ NPs–folic acid–ICG (PS–CS–Au–Fe_3_O_4_–FA–ICG) all together formed nanocomposites, which have a good PTT effect under NIR irradiation. Chitosan is a biodegradable, biocompatible, and non-toxic natural sugar biopolymer. AuNPs act as a CT imaging probe that can induce a strong X-ray attenuation. Fe_3_O_4_ NPs are used as MRI agents in clinical application. FA, used to target folic acid overexpressing cancer cells for the therapy of tumors, can enhance uptake by cancer cells. ICG acts as a photosensitizer. HeLa tumor-bearing mice were used for *in vivo* studies. The nanocomposites can be delivered in a targeted manner to the cancer site and tumor growth could be reduced and even be completely ablated under 808 nm laser irradiation,^103^ see Fig. 9b and c.

In another case, gold nanostars (AuNSs), PDA, polyethyleneimine (PEI), and FA make an APP nanocomposite. AuNSs have good biocompatibility and PDA increased the stability through laser exposure, and APP–ICG also acts as a contrast agent. MCF-7 cell lines were used for cell viability assay. These AuNS–PDA–PEI–FA components enhanced the PTT and PDT effect under NIR irradiation. The APP nanocomposite has some unique features such as good biocompatibility, active targeting ability, excellent loading, and protection ability for negatively charged molecules.^104^ AuNRs and AuNPs have PDT and PTT effects to destroy malignant or tumor cells and are conjugated with the hydrophilic photosensitizer ICG to make the AuNRs–AuNPs–ICG composite for attaining PDT and PTT. A human lung carcinoma malignant cell line was (A549) used for *in vivo* studies.^29^ Li and group reported that GNRs are a bright contrast agent for imaging and PTT. Mesoporous silica was coated on the GNRs by hydrolysis to form the GNRs–SiO_2_ compound. Finally, ICG was incorporated into the compound, and the final product is GNRs–SiO_2_–ICG, which showed enhanced stability and biocompatibility, and a superior antitumor effect. The human melanoma cell line A375 was used for *in vitro* cell cytotoxicity studies.^105^ ICG (hydrophilic) and GNPs (hydrophobic) were combined together to make ICG–GNP nanoclusters. They have both PAI and PTT properties. These nanoclusters provide heat for PTT and eradicate tumors under NIR irradiation. Mainly this complex is cytotoxic to triple-negative breast cancer (TNBC) cells. In this study, mouse 4T1 mammary carcinoma cells were selected for *in vitro* studies. Laser irradiation reduced the tumor volume and controlled the growth under 808 nm. Furthermore, ICG-loaded Au–SiO_2_–mSiO_2_ has been designed to achieve high PTT and PDT performance. Au–SiO_2_–mSiO_2_ NPs act as the carrier. The photothermal effect of AuNRs improved the total PTT effect. The mesoporous silica layer served as the porous host for ICG and protected ICG from oxidizing by singlet oxygen. The dense silica layer, which acted as the insulator between the AuNRs and ICG, loaded in the mesoporous silica layer, avoided the photobleaching of ICG from direct contact with AuNRs. The human liver carcinoma cancer cell line HepG2 was used and destruction occurred by heat.^106^ Moreover, ICG was conjugated with mesoporous silica and coated with gold nanobipyramids (GNBs) to make the composite GNB@SiO_2_–ICG, which was used for PA and PTT. In this composite, GNBs were used for PA imaging and PTT. ICG was used for fluorescence (FL) imaging, PA, and PTT. A375 cells were used for *in vitro* studies under 808 nm NIR irradiation.^107^

### 4.3. Mesoporous silica nanocarriers

**Fig. 10:**
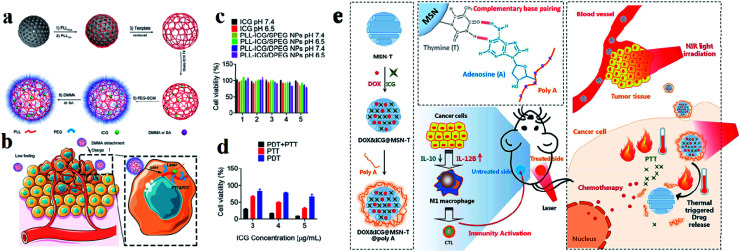
Preparation of (a) PLL–ICG/DPEG and (b) PLL–ICG/SPEG NPs using the MS templating method for PTT and PDT. (c) Cell viability in the presence of ICG, PLL–ICG/DPEG NPs, and PLL–ICG/SPEG NPs with NIR laser irradiation. (d) Comparison of PDT, PTT, and simultaneous PDT/PTT effects on cell viability. Reproduced with permission from ref. 115. Copyright 2019, American Chemical Society. (e) Schematic illustration of the DOX&ICG@MSN-T@poly A design, triggered drug release, anticancer process, and immunity activation mechanism. Reproduced with permission from ref. 116. Copyright 2019, American Chemical Society.

Among various inorganic nanomaterials, mesoporous silica NPs (MSNs) are an important class of porous materials that have colloidal stability. They have attracted great attention for use in drug delivery systems for targeted cancer treatment and have high potential for use as multifunctional drug delivery nanocarriers.^108^ Mobil composition of matter (MCM)-41 is the first-ever reported well-ordered crystalline molecular sieve in the early 1990s. The formation of MSNs is mainly based on the assembly of a surfactant and silica species through co-condensation, as well as particular electrostatic interactions between the organic surfactant template (CTAB) cetyl trimethyl ammonium bromide and silica.^109^ For improving the drug loading efficacy and specific targeting capacity, asymmetric mesoporous silica-based nano-architectures, referred to as Janus NPs, have also been designed recently to enhance the intrinsic functionalities of MSNs for providing dual drugs and improving compatibility.^110,111^ These NPs have well-defined and tunable porosity on the nanometer scale, high loading capacity, and multiple functionalities for targeting and entering different types of cells.^112^ Generally MSNs are internalized into cells through endocytosis and have some distinct advantages such as tunable pore size which is used for controlled drug release, high surface area and large pore volume supporting a high payload of drugs, a tunable particle size, facile surface functionalization benefiting targeted delivery, a flexible nano-structure, excellent biocompatibility, and biodegradability.^39^ Hollow MSNs have been used to enhance the loading capacity of various anti-metastatic drugs and genes, such as silibinin, DOX, and siRNA, extensively improving their anti-metastatic efficacies.^113^ Silica was approved by the US FDA for biomedical applications. Arginine–glycine–aspartic acid (RGD) was conjugated with MSNs and again loaded with ICG dye. MSNs act as drug nanocarriers in cancer therapy, and have unique properties, including high chemical stability, controllable pore size, large surface area, and excellent drug loading ability. Synthesized RGD was conjugated with MSN NPs and loaded into ICG to make the compound ICG–MSNs–RGD. RGD is overexpressed in many tumors and is a peptide ligand for the integrin αvβ_3_ receptor.^114^ Dual responsive polypeptide NPs were loaded with ICG with the help of mesoporous silica by a templating method for both PTT and PDT. The HeLa cell line was used for cell cytotoxicity studies. Polypeptide NPs composed of thiol-modified polylysine (PLL), which is cross-linked with disulfide bonds and modified with PEG and pH sheddable dimethyl maleic anhydride (DMMA), exhibit reduction and pH-responsiveness. ICG loaded with MS and DMMA modified PLL NPs show good thermo responsiveness and singlet oxygen generation under laser irradiation, which induces higher cytotoxicity against cancer cells compared to succinic anhydride (SA) modified PLL NPs,^115^ see Fig. 10a–d. Accordingly, the prepared MSNs, DOX, and ICG were co-encapsulated inside MSN-T (thymine modified MSNPs) to obtain DOX–ICG–MSN-T. Poly A was used for ceiling the pores of MSNs rapidly through A–T pairing (DOX–ICG–MSN-T–Poly-A) to avoid drug release during the nanomedicine delivery process in the blood because the pairing is constant at physiological temperature. After these compounds arrive at the tumor tissue, an NIR laser was used to irradiate the tumor tissue (808 nm). The photothermal conversion ability of ICG heated and induced the breaking of the A–T pairing and triggered DOX release enabling chemotherapy activity against cancer cells and induced cancer cell death,^116^ see Fig. 10e.

A structure with an upconversion NP (UCNP) core and a mesoporous silica (mSiO_2_) shell was prepared, whose matrix was loaded and sealed with a high concentration of ICG molecules. This combination of UCNP–mSiO_2_–ICG has some unique features: (1) the UCNP core produces efficient green and red upconversion luminescence for optical imaging; (2) the ICG in the mesoporous silica shell enables both PAI and PTT. It could produce a sufficient amount of heat causing hyperthermia to kill cancer cells under 800 nm laser irradiation.^117^ An ideal system with MSNs and ICG covalently conjugated and loaded with the anticancer drug mitoxantrone (MTX) was developed, and was used as an innovative photoacoustic probe. MTX is an anthraquinone derivative drug widely used for treating breast and prostate cancer. ICG–MTX is used in both PAI and PTT under NIR irradiation.^118^ Another report demonstrated that ICG is encapsulated inside the pores of PEGylated MCM-41 MSNs (ICG–MSNs). Compared to free ICG, ICG–MSNs show greater stability and decreased toxicity, and the photoacoustic effect increases nearly 400% due to the high photothermal conversion of the encapsulated dye molecules. MSNs are highly stable and adaptable; a PAI probe was prepared by loading ICG dye inside the pores of PEGylated MCM-41 MSNs to increase both the stability of the dye and its photoacoustic effect.^119^ Further, pH responsive ICG loaded with zwitterionic fluorescent carbon dots (CDs) was encapsulated in MSNs for PTT. ICG was loaded into the MSNs through hydrophobic and electrostatic interactions between zwitterionic CDs and ICG. After loading the ICG, the porosity of the MSNs was altered because of the intermolecular interactions between the CDs and ICG inside the MSN shell and core. The photothermal conversion of ICG-MSN (CD) showed a sufficient amount of heat generation to kill cancer cells at an acidic pH. ICG-MSN (CD) resulted in good cell viability of MDA-MB-231 cells with no irradiation and high necrosis was also observed.^120^ Besides, coraline is a type of alkaloid used in cancer chemotherapy; it can not only be loaded into the pores of MSNs but also bind poly (A). Both coraline and ICG were loaded into adenine DNA poly (A) functionalized MSNs. ICG and one more cargo were co-loaded into MSNs, which showed high efficiency for the conversion of NIR light into heat, which can provide the basis of hyperthermal therapy and help pore opening upon NIR irradiation. *In vitro* studies using human hepatoma (HepG-2) cells demonstrated that this system could perform well under NIR irradiation, for controlled drug release and chemothermal cancer treatment.^121^ PEG-coated MSNs were used as the carrier and they encapsulated ICG and DOX. A tumor cell targeting motif (RGD) was decorated on the MSNs through a host–guest interaction between cyclodextrin (CD) and adamantine. PEG chains can protect the drug-loaded MSNs during circulation. MMP-2 is overexpressed in tumor tissues. Murine mammary carcinoma (4T1) cells were used for *in vitro* studies and BALB/c mice were used for *in vivo* studies. This combination (PEG–MSN–ICG–DOX) was used in chemotherapy and PTT.^122^ Another study reported that ICG is loaded into hollow MSNPs (ICG–HMSNP) as an activatable theranostic platform. In the extracellular region, ICG–HMSNPs were nonfluorescent and non-phototoxic. After the NPs entered the cancer cells through endocytosis, they became highly fluorescent and phototoxic. ICG loaded HMSNPs (ICG@HMSNP) were used as an activatable theranostic agent for cancer.^123^ Ding and colleagues demonstrated the incorporation of ICG with bismuth selenide NPs (BS NPs) to make the nanocomposite PM@BS–ICG NPs to improve ICG stability in the drug delivery system. Most importantly, the BS NPs inhibited hyperthermia under NIR irradiation and enhanced better antitumor activity.^124^

### 4.4. Calcium phosphate nanocarriers

**Fig. 11:**
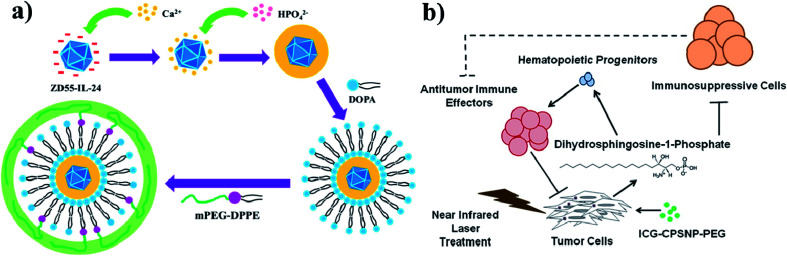
(a) Synthetic route used to prepare PLC-ZD55-IL-24 and graphical representation of tumor volume increase without NIR irradiation. Reproduced with permission from ref. 127. Copyright 2016, American Chemical Society. (b) Schematic illustration of PhotoImmunoNanoTherapy utilizing ICG–CPSNP–PEG, which exerts an antitumor effect by generating dihydrosphingosine-1-phosphate (dhS1P). Reproduced with permission from ref. 128. Copyright 2013, American Chemical Society.

Calcium phosphate (CaP) is an inorganic substance and acts as a nanocarrier for drug delivery systems. Due to their high biocompatibility, nontoxicity, pH-responsiveness, and bioactivity, CaP-based NPs are considered as important theranostic agents. CaP occurs naturally in teeth and bones in the human body. Even CaP-based nanomaterials have some drawbacks such as complicated synthetic steps, large size, and obvious aggregation. Their enhanced PTT with chemotherapy can improve anti-cancer efficiency.^125^ CaP is a highly biocompatible inorganic biomaterial and readily combines with adenovirus, enhancing adenovirus-mediated gene transfer. Amorphous CaP is synthesized using aqueous solutions of calcium and phosphate ions. CaP rapidly dissolves under acidic pH conditions and is endocytosed by cells. CaP NPs are used to encapsulate chemotherapeutic drugs and deliver them to the tumor site,^126,127^ see Fig. 11a. Further, in one more study, ICG and calcium phosphosilicate nanocomposite particles (CPSNPs) were evaluated in murine models of breast cancer, pancreatic cancer, and metastatic osteosarcoma for PDT. ICG–CPSNPs were coated with PEG to form a nano complex ICG–CPSNPs–PEG, which acts as a theranostic agent for cancer,^128^ see Fig. 11b.

To better understand, PDA-integrated mesoporous calcium phosphate (mCaP) inorganic hollow JNPs (HJNPs) were decorated with PEG–SH and ICG (PEG–ICG–PDA–mCaP-HJNPs); however, the other mCaP sides were utilized as storage spaces for anti-cancer drugs. The synthesized nanomixture shows good drug storage capability, outstanding PTCE, NIR and pH dual responsive drug release properties and PAI for photothermal cancer therapy.^129^ Furthermore, the ICG fluorophore with efficient NIR emission was embedded into the CPNPs. These CPNPs were further coated with carboxylate or PEG. CPNPs have better optical properties than free fluorophores. These ICG CPNPs were used for both *in vivo* studies and PTT of cancer and also used in sensitive diagnostic imaging applications.^130^ The study carried out with ICG alone has some restrictions, and to overcome these hurdles, ICG was encapsulated into the CPSNPs and used for PDT. The ICG loaded CPSNPs act as photosensitizers in leukemia. Leukemia is a type of cancer that occurs in the blood or bone marrow. The specific and selective targeting of ICG–CPSNPs would allow successful PDT of leukemia. They showed minimal side effects.^131^ It is worth noting that the CPNPs can target breast and pancreatic cancer lesions. The NIR imaging agent ICG is embedded into the CPNPs, which are 20 nm diameter composites composed of an amorphous calcium phosphate matrix doped with silicate. Avidin is conjugated with CPNPs (avidin CPNPs) which permits the targeting of transferrin receptors, which are highly expressed on breast cancer cells. Likewise, PEG-coated CPNPs permit targeting of gastrin receptors, which are overexpressed in pancreatic cancer lesions.^132^ CPNPs act as a trimodal contrast agent for combined optical, magnetic, and nuclear imaging, and their *in vivo* application has been demonstrated using mouse models. Calcium phosphate NPs (nCP) and ICG were doped with gadolinium (Gd3þ) and surface labeled with technetium 99m (Tc) methylene diphosphonate (MDP). These three contrast properties might be derived from a single NP. This trimodal contrast agent is multifunctional (MF-nCP). PEG also acts as a capping agent.^133^

### 4.5. Layered double hydroxide nanocarriers

**Fig. 12:**
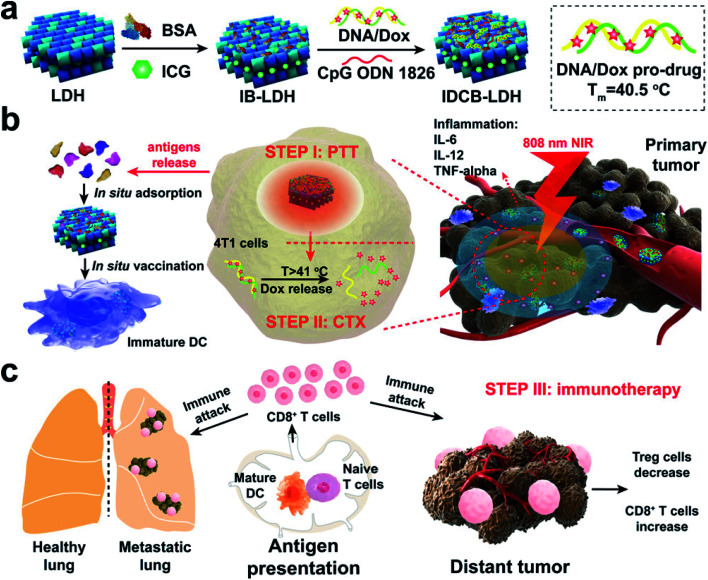
Schematic representation of a multifunctional IDCB–LDH nanomedicine. (a) The hybrid nanomedicine is constructed by first coating with BSA and then orderly loading ICG, DOX/DNA prodrug, and C_P_G ODN 1826. (b) IDCB–LDH with 808 nm NIR irradiation heats the tumor tissues and releases DOX at a temperature above 41 °C to kill tumor cells through efficient PTT. (c) Mature DCs activate naïve T cells in dLNs and induce potent CTLs. Reproduced with permission from ref. 135. Copyright 2019, American Chemical Society.

In nanotechnology, layered double hydroxides (LDH) have some unique features such as controllable size and morphology, good biocompatibility, great chemical stability, and ion-exchange properties for drug loading and release. Due to these properties, LDH vehicles could be designed as smart delivery systems to transport various drugs, antibodies, and enzymes. Most importantly, LDHs have also been reported as promising agents for ICG co-delivery. Among the inorganic nanomaterials studied, LDHs are considered as biocompatible nanostructures and have received considerable attention in past years.^134^ Also, LDH NPs are unique platforms to construct such multifunctional nanomedicines cost-effectively. Meanwhile, LDHs have high affinity for proteins, DNA, and siRNA as well as small drug molecules. In this work, the authors mainly integrated ICG, DOX, and adjuvant CpG into a LDH for combined PTT, chemotherapy, and immunotherapy,^135^ see Fig. 12.

Wei and colleagues demonstrated the incorporation of LDH NPs and ICG for organ-specific targeting, and the final nanocomposite LDHs–NH_2_–ICG was further coated with chitosan molecules. Chitosan-coated LDHs–NH_2_–ICG was confirmed by imaging and the composite has high biocompatibility, high fluorescence intensity, high organ-specific targeting, low cytotoxicity and increased stability.^136^ In another case, Wei and co-workers constructed chitosan-coated LDH NPs loaded with ICG with photoactive properties for PDT. Chitosan is an efficient positively charged polymer, which is used to achieve photodynamic efficacy, and chitosan-coated LDH NPs may increase the photosensitizer excitation efficiency.^137^ Wang and colleagues designed an ultrathin Ce doped Cu–Al LDH and ICG loaded nanocomposite. The synthesized ICG/CAC-LDH was used as a photothermal agent.^138^

## 5. ICG-based hybrid nanocarriers

**Fig. 13:**
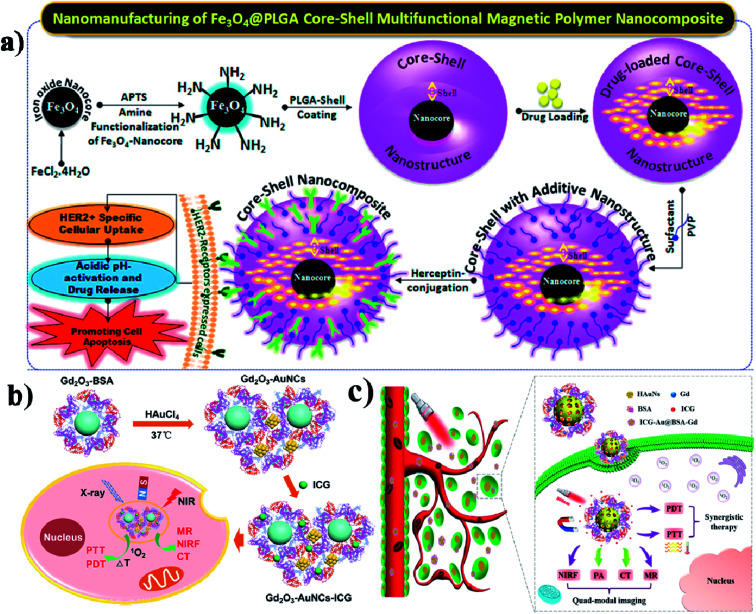
Schematic illustration of the synthesis of Fe_3_O_4_@PLGA–PVP core–shell NCs for HER2 targeted endocytosis and breast cancer application of the nanocomposite. Reproduced with permission from ref. 148. Copyright 2016, American Chemical Society. (b) Schematic illustration and preparation of gadolinium oxide–gold nanocluster nanohybrids for multi-modal imaging therapy. Reproduced with permission from ref. 149. Copyright 2017, American Chemical Society. (c) Schematic illustration of the ICG–Au@BSA–Gd theranostic agent for PTT and PDT effects for cancer therapy. Reproduced with permission from ref. 150. Copyright 2017, American Chemical Society.

Due to their physicochemical properties, nanohybrids have attracted much attention in bio-related systems recently. And nanohybrids are composed of organic and inorganic components and they could be found in nature such as in mollusk shells and teeth. Further, hybrid nanohybrids have many biomedical applications including drug delivery, gene delivery, phototherapy, and imaging.^139^ In this study, the authors developed a composite for intracellular cancer drug delivery. Most importantly, cystamine-integrated periodic mesoporous organosilica (Cys-PMO) hybrid NPs were developed, and after that Dox anticancer drug was loaded. DOX-loaded Cys-PMO nanohybrids show better cytotoxicity in the tumor environment.^140^ Lipid–polymer hybrid NPs (LPHNPs) consist of a polymeric core and are coated with single or multiple layers of a lipid shell, and have many advantages. The LPNPs show high stability, controlled release of drugs, high biocompatibility, and reduced leakage of hydrophobic drugs.^141^ Garg and group reported that methotrexate (MTX) and aceclofenac (ACL) could be co-encapsulated in LPHNPs. These nanohybrids are used for target-specific and controlled release of the drugs against breast cancer.^142^ In another case, Xi and co-workers synthesized Au–PLGA hybrid NPs, to which PDA was also added, which acted as a bridge to connect Au and PLGA, and finally, PEG acted as a hydrophilic shell and Au–PLGA NPs served as a hydrophobic core (PLGA/DOX@PDA–Au–PEG NPs). These Au–PLGA nanohybrids show several advantages such as high photothermal conversion efficiency, heat-triggered drug release in the tumor site, moderation of the tumor environment, and generation of a high level of ROS to kill the cancer cells.^143^ Hong and co-workers developed PEG-coated zinc oxide nanorods (PEG–ZnO NRs) with piperlongumine (PL) for targeted chemo and photodynamic combination therapy.^144^ Accordingly, Hayashi and group synthesized fluorophore–siloxane hybrid NPs (HNPs) with porphyrin incorporated into HNPs through a covalent bond, and at last, ICG was coated on the HNP surface. Furthermore, the HNPs allow tracking of the immune cells in the whole body.^145^ Wu and group formulated an AuNCs/PPI–ICG nanohybrid for dual-modal imaging for photothermal and photodynamic therapy. PPI (pea protein isolate) and ICG were loaded onto AuNCs, which show synergistic therapeutic effects on cancer cells *in vitro*.^146^ Here the authors reported temperature-sensitive hybrid bicelles which were used to encapsulate hydrophobic DOX and ICG and make nanohybrid DOX/ICG@HBs. These nanohybrids show better photostability when ICG is encapsulated into the lipid bilayer membrane than free ICG.^147^ Vivek and group prepared a nanocomposite by coating an iron nanocore and a hydrophobic PLGA polymer nanoshell, which is used to encapsulate a drug. Tamoxifen is an anticancer drug that was packed into the nanocomposite. Further, the nanocore was stabilized by poly-(vinylpyrrolidone) (PVP) for targeting the HER2 ligand. The final Her-Fe_3_O_4_@PLGA-PVP nanocomposite is used to promote HER2 receptor-mediated endocytosis and it induces apoptosis,^148^ see Fig. 13a.

In this work, protein stabilized gadolinium oxide (Gd_2_O_3_–AuNCs) gold nanoclusters were constructed, and used for multimodal imaging and drug delivery. This nanohybrid was developed by integrating Gd_2_O_3_ nanocrystals and AuNCs into BSA as a stabilizer,^149^ see Fig. 13b. Similarly, You and colleagues combined gadolinium (Gd) based BSA and hybrid coated gold nanoshells (Au) together to make the Au@BSA–Gd nanohybrid multifunctional drug, and it has good PTT, CT, and PA activity. Most importantly nanohybrids act as a T1 contrast agent for MRI imaging,^150^ see Fig. 13c.

## 6. Conclusion and future outlook

Cancer is a group of diseases involving abnormal cell growth with the potential to invade and spread to other parts of the body. Cancer treatments include chemotherapy, radiation therapy, and surgery. Nanotechnology-based treatment is also used in the form of nanomedicine to reduce or control tumors. As we discussed in the earlier segments, there have been many studies on ICG formulated nanotherapeutics in recent years. We have reviewed many papers describing the development and use of the fluorescence contrast agent ICG in cancer treatment. In this review, we systematically bring together the recent developments and progress of ICG nanotherapeutics in multimodal cancer therapy. Even though the fabulous advancements in constructing an ICG mediated drug delivery system have offered favorable results in the past decades, still ICG suffers from some challenges in cancer imaging and therapeutics. In biomedical applications, ICG has efficient fluorescence intensity and PA imaging ability. A combination of photothermal and photodynamic therapy with chemotherapy and immunotherapy has been evolved for different cancer treatment strategies. With the prompt improvement of organic, inorganic, and hybrid nanomaterials, the construction of NPs with ICG has made great progress. In the meantime, other approaches are also welcome as simple and superficial methods. Recently, the fundamental advantages of ICG, including outstanding NIR fluorescence imaging capability and good biocompatibility, made ICG an outstanding agent for the diagnosis and treatment of cancers. In this review, we have reviewed many papers and characterized the enhancement and usage of the fluorescent contrast agent ICG in many applications. At the same, ICG had to be excluded from many studies due to the limitations such as photodegradation, thermal degradation, aqueous instability, short circulation time, rapid clearance, photobleaching, and low fluorescence quantum yield. For better efficacy, ICG could be loaded or encapsulated, or coated on NPs. ICG loaded NPs have some unique characteristics such as high photostability, long circulation time, thermal stability, tumor-targeting ability, low toxicity, and high tumor ablation efficiency. Also ICG acts as an efficient phototheranostic agent in cancer treatment. Therefore, to meet the growing need for improved cancer therapy, ICG can effectively be used as an alternative both as a photosensitizer and a credible therapeutic agent. Due to the limitation of half-life, ICG requires incorporation with NPs, which can be further studied to produce efficient therapy shortly. It is essential to explore how ICG and ICG formulated nanocarriers behave in the tumor site, including their biodistribution, potential degradation, clearance, and biocompatibility *in vivo*. In conclusion, emerging ICG platforms could be offered as unique tools for image-guided and antitumoral therapy. On the other hand, we need to construct different ICG-based nanocarriers to try and find the right strategy. Therefore, the deep study of the application range of ICG-based delivery systems will be constantly extended towards the future. Although many studies on ICG-loaded carriers have been published in past years and promising results have been achieved, further studies are essential to extend their use for human clinical applications.

## Abbreviations

NPs: Nanoparticles
nm: Nanometers
ICG: Indocyanine green
NIR: Near-infrared region
US FDA: United States Food and Drug Administration
PDT: Photodynamic therapy
PTT: Photothermal therapy
PLGA: (Polylactic-*co*-glycolic acid)
PEG: Polyethylene glycol
PCA: Photothermal coupling agent
NILI: Near-infrared laser illumination
LAAA: Light-activated anti-bacterial agent
PLL: Poly-L-lysine
FA: Folic acid
DLS: Dynamic laser scattering
TEM: Transmission electron microscopy
CBP: Carboplatin
MDR: Multidrug resistance
DOX: Doxorubicin
HER2: Human epidermal growth factor receptor 2
PLA: Poly(lactic acid)
HA: Hyaluronic acid
CD44: Cluster determinant 44
NIRF: Near-infrared fluorescence imaging
HANPs: Hyaluronic acid nanoparticles
RGD: Arginine–glycine–aspartic acid peptide
PCE: Photothermal conversion efficiency
EPR: Enhanced permeability and retention
TNBC: Triple-negative breast cancer
PCL: Poly(caprolactone)
mAb: Monoclonal antibody
HDLs: High-density lipoproteins
NLC: Nanostructured lipid carriers
PTX: Paclitaxel
PEO: Polyethylene oxide
PAMAM: Polyamidoamine dendrimer
NGs: Nanogels
EEs: Encapsulation efficiencies
PD: Polyacylthiourea dendrimer
PET: Positron emission tomography
SPIO: Superparamagnetic iron oxide
PB: Prussian blue
PAI: Photoacoustic imaging
MRI: Magnetic resonance imaging
DTPA: Diethylenetriamine pentaacetic acid
Fe_3_O_4_: Magnetic iron oxide
PFP: Perfluoropentane
CCM: Cancer cell membrane
PDA: Polydopamine
mGO: Magnetic graphene oxide
APT: Aptamer
AuNPs: Gold nanoparticles
GNR: Gold nanorods
AuNCs: Gold nanoclusters
mPEG: Poly mermethoxypoly(ethylene glycol)
ER: Endoplasmic reticulum
Hb: Hemoglobin
ICD: Immunogenic cell death
CT: Computed X-ray tomography
LA: Lactobionic acid
PS: Polystyrene
AuNS: Gold nanostar
PEI: Polyetherimidefolate
MSNs: Mesoporous silica nanoparticles
PLL: Polylysine
DMMA: Dimethylmaleic anhydride
SA: Succinic anhydride
UCNP: Upconversion nanoparticle
CD: Carbon dot
CD: Cyclodextrin
HMSNP: Hollow mesoporous silica nanoparticle
CaP: Calcium phosphate
mCaP: Mesoporous calcium phosphate
CPNPs: Calcium phosphate nanoparticles
CPSNPs: Calcium phosphosilicate nanocomposite particles
nCP: Calcium phosphate nanoparticles
Gd: Gadolinium
Tc: Technetium
MDP: Methylene diphosphonate.

## Conflicts of interest

The authors declare no conflict of interest.

## Supplementary Material
